# Psychiatrists' use of formulation: Commentary on … Psychiatrists' understanding and use of psychological formulation^†^

**DOI:** 10.1192/pb.bp.115.052746

**Published:** 2016-08

**Authors:** Patricia M. Hughes

**Affiliations:** 1 St George's, University of London

## Abstract

Both psychologists and psychiatrists are trained to write formulations of their patients' illnesses, with some differences in how they do this. Psychologists focus on psychological understanding, while psychiatrists' formulation brings together aetiology, functioning and a management plan. Mohtashemi et al's study records how some psychiatrists understand formulation and its usefulness. Time pressure was an important barrier to making a full formulation, and some believed the medical role of the psychiatrist was a priority. The study illustrates some of the challenges facing psychiatrists working in the NHS in terms of maintaining high clinical standards and a holistic approach to patient care.

For most of my career, good clinical practice for a psychiatrist seeing a patient for the first time was to make a full assessment and outline a management plan, then extend the plan to include the views of other members of the team, including the psychologist, if the team was lucky enough to have one. And once upon a time, long, long ago, there was also a social worker and an occupational therapist; this is now a distant memory. I was engaged by the lively picture of current mental health practice described by Mohtashemi and colleagues,^1^ although I did find some of it rather depressing. So while the paper is titled ‘psychiatrists’ use of formulation', some explanations for non-use are equally important. This is a relevant sub-theme, touching on the real difficulties psychiatrists working in the National Health Service (NHS) now have in finding time to maintain some of the standards of good practice. The daunting mountain of work and information-gathering demanded of doctors is in danger of obliterating the reflection, the discussion of possibilities, and the holistic approach that makes our specialty so worthwhile and intellectually challenging.

The paper derives from a research project for a clinical psychology doctoral thesis, and offers a fresh view of psychiatric practice. Guidelines on how to write a formulation are published by the British Psychological Society^2^ and by the Royal College of Psychiatrists.^3^ The former strongly emphasises psychological formulation, while that for psychiatrists in training is broader, summarised in a recent editorial in the *British Journal of Psychiatry* as a balanced evaluation ‘that brings together aetiology, severity and functioning and should lead to a management plan’.^4^ The researchers argue that what was missing from the assessment of most psychiatrists was a psychological formulation, and they may be correct, but I suggest there is a semantic gap here: there is a wide range of views on what constitutes a psychological formulation or a psychological component of a formulation. Until recently, I was an NHS consultant psychiatrist in psychotherapy and I am psychoanalytically trained. The psychological formulation that I, as a specialist, would have expected to provide would have a different emphasis from that of a trainee psychiatrist or a community mental health team (CMHT) consultant psychiatrist. While I might have offered something closer to that of my psychology colleagues, the model for psychiatrists in acute services would include a broad assessment, and incorporate factors that contribute to diagnosis and to the development of a care plan. In my experience, the vast majority of experienced general adult psychiatrists, or specialists in other acute psychiatric settings, recognise the psychological or developmental component of a formulation, but it would be only a part of a larger and broader formulation. The researchers' view was that the psychiatrists interviewed were less certain than their psychology colleagues about what a formulation ought to include: if this is the case, then our training and the MRCPsych exam are failing their purpose. Surely at some stage in their training, psychiatrists will have to be signed off or examined as making a satisfactory assessment, thinking about what triggered the illness or episode, evaluating how severe it is, finding out something about usual functioning and social support, thinking about personality, and from these producing a management plan?^3^ And yet, there was indication in Mohtashemi *et al*'s paper that in real life a busy psychiatrist may not have time for these luxuries.

Data analysis indicated that psychiatrists see diagnosis as ‘the foundation of their role, prioritised alongside medication’. But if the formulation is to help diagnosis, is there any conflict here? On the other hand, many psychiatrists, indeed, arguably all good doctors, believe that patient care goes beyond a diagnostic category, and that good care needs a wider understanding of the context and other factors that can help or impede recovery. The authors appear to be concerned that if psychiatrists do not formulate in a more comprehensive way, then practice will become, or may have already become, a dichotomy of ‘psychiatric diagnosis versus psychological formulation’. Bracken and colleagues' plea^4^ is to recognise the place of ‘relationships and values’ in mental healthcare, in a climate which, in their view, idealises a neuroscience model. Mohtashemi and colleagues see the apparent increased emphasis on diagnosis as the doctor's prime role, as a symptom of this medicalisation of healthcare, and some of their interviewees' comments seem to support this. One doctor says ‘if someone is bipolar [they're] bipolar […] you don't need to formulate … you do a diagnosis’. Others felt medication was not the best approach for a particular patient, but time constraints meant that they had to ‘come across as doing something’.

That brings us to the most interesting, and disquieting, aspects of the paper, ‘Barriers to formulation’, and ‘Making a Frankenstein monster’. The researchers note that ‘a large amount of time [in interviews] was spent in talking about […] the politics surrounding psychiatry and limitations within NHS services.’ How familiar. Of more concern, however, was that ‘participants perceived themselves to be faced with multiple barriers that affected their ability to formulate and think reflectively.’ One interviewee complained of being under ‘immense pressure’ to make quick decisions in assessing risk and making a diagnosis, which did not allow for ‘reflective practice’. Of course, sometimes a quick decision is essential, usually with an acutely ill patient; it does not mean no subsequent formulation, nor does it mean that treatment options are not discussed at some point with the patient. It was a pity that we did not know how many participants were trainees, and how many were more experienced doctors. I imagine that psychiatrists with greater experience can balance core tasks such as risk assessment and diagnosis with a richer assessment of the patient's problems and management needs. Consultants have learned to be efficient with their time. Although I was a trainee in a very different health service, more than 30 years ago, I do recognise some of the experience described by the interviewees. I was slow. It took forever to write letters, which were ridiculously long. I saw patients more often than was necessary, because I was afraid of missing something, spent weeks preparing for presentations. But there did seem to be time to think about patients, speak to colleagues, and explore the factors contributing to the patient's illness. I do not remember that we were ever under pressure to make a quick diagnosis and prescribe unless there was a clear clinical indication that rapid intervention was important.

Reassuringly, most participants in the study perceived professional rivalry as unhelpful, and wanted to see ‘a […] process of […] integration of psychologists and psychiatrists’. The researchers believe different disciplines have specialist skills and that the psychiatrist and the team should use the skills of the psychologists where they can. Participants in the study overall supported this. Some, however, are reported as seeing some psychologists as anti-psychiatry. How unwise, when there is so much work to do. One would imagine anyone with any sense at all would welcome another pair of able hands. On balance, the impression was that psychiatrists value psychological understanding, and value the skills of psychologists. Yet how frustrating to know that your patient could benefit from psychological treatment, but resources are slender, waiting lists long, and the immediate imperative is to get the patient treated and ‘off the books’. As the paper remarked, certain services, such as forensic, must formulate: management is long term, patients are high risk and publicly so, and every resource must be called upon. In contrast, most community services are swamped with work, are constantly threatened with yet more cuts, and are primarily alert to patient safety and to containing workloads. Participants suggested that psychologists could train nurses in basic formulation skills, implied as psychological formulation. Interesting that they did not suggest psychologists train psychiatrists – I would guess psychiatrists generally believe their ability to understand psychological issues is not very different from psychologists, but consider they cannot devote time to offering psychological treatment.

Oddly, no one mentioned the demands of the online data recording. In my latter years as a consultant this occupied a huge amount of time, compared with earlier, less intensively regulated recording of patient information. This must be a generation that has grown up with this expectation and knows no different. And perhaps this is also a disincentive to write a formulation, as it will be swallowed by a database, out of sight and not so readily accessed as case notes were in their day. And no one mentioned the relentless demand for mental health tribunals, another painful drain on clinical resources.

This paper was written in an NHS where money is tight and getting tighter. Managers have to make savings, and at the coalface, psychiatrists must see patients as quickly as possible, and must offer some kind of intervention. It is not good medicine, however, if being seen to do something, even if inappropriate, is accepted by practitioners as adequate. Nor is it good management if a service is selected to be axed because it will save exactly the sum needed, or because the waiting lists for the service are long. These examples are both true. Annual ‘cost improvements’, seeing more patients for less money, replacing senior psychologists with junior ones, doctors with nurses, trained nurses with healthcare assistants, must in the end lead to a different and inferior service which may not be more cost-effective. We will be denying reality and indulging in omnipotent fantasy if we allow ourselves to think that we can do the same with fewer resources; we are dishonest if we claim that things are getting better and better, when in reality they are struggling to remain half decent.

What is the future for psychiatrists? Will the next generation of psychiatrists be permitted to do only what no one else is allowed to do? Diagnosis and prescription, mental health tribunals, arguing for resources, and maintaining existing good services. We may be thought too expensive to be allowed to spend more than a few minutes with individual patients. Postgraduate training may be limited to skills approved for psychiatrists, perhaps more towards the pharmacological end of the spectrum than the psychological, so a skill such as family therapy may be reserved for less costly colleagues. We will learn about management, for this will be a crucial interface in organising and planning our services. What an impoverished career!

However, I am an old psychiatrist not a young one, and shroud waving is a temptation for the old. So let us look at the positives before I end. My experience in the latter part of my career of most trainees entering psychiatry filled me with hope for the future of our specialty. And despite the complaints, and the real worry about lack of time to practise good psychiatry, the doctors interviewed for this paper sounded like people who did think, who found time to speak to the researchers, and while not happy with the present system, could see what was wrong and how it could be improved. They cared about their patients, and were articulate and imaginative. I hope they fight for the right to give patients the holistic care that they deserve.

And the young psychologist whose research was the basis of the paper completed a useful piece of work, which must have needed perseverance, patience and original thought. The future may not be hopeless.
